# Chemical Composition of Essential Oil and Leaf Anatomy of *Salvia bertolonii* Vis. and *Salvia pratensis* L. (Sect. *Plethiosphace*, Lamiaceae)

**DOI:** 10.3390/molecules14010001

**Published:** 2008-12-23

**Authors:** Goran Anačkov, Biljana Božin, Lana Zorić, Dragana Vukov, Neda Mimica-Dukić, Ljiljana Merkulov, Ružica Igić, Marina Jovanović, Pal Boža

**Affiliations:** 1 Faculty of Natural Sciences, Department of Biology and Ecology, Trg D. Obradovica 2, Novi Sad, Serbia; E-mails: ganackov@ib.ns.ac.yu (G. A.), lana@ib.ns.ac.yu (L. Z.), merkulovlj@ib.ns.ac.yu (L. M.), igicr@ib.ns.ac.yu (R. I.), dvukov@ib.ns.ac.yu (D. V.), bozap@ib.ns.ac.yu (P. B.),; 2 University of Novi Sad, Faculty of Medicine, Hajduk Veljkova 3, Novi Sad, Serbia; E-mail: brkics@uns.ns.ac.yu (M. J.); 3 University of Novi Sad, Faculty of Natural Sciences, Department of Chemistry, Trg D. Obradovica 2, Novi Sad, Serbia; E-mails: mimica@ih.ns.ac.yu (N. M-D.)

**Keywords:** *Salvia*, Essential oil, Micromorphological characters, Taxonomic relationship.

## Abstract

The taxonomical relationship between *Salvia pratensis* and *S. bertolonii* has been unclear for a long time. *Salvia bertolonii* has alternatively been considered a synonym, a subspecies, a problematic subspecies and a form of *Salvia pratensis*. However, both these two species are sometimes used in traditional medicine instead of sage (*Salvia officinalis*) or as an adulteration for the same drug. In order to confirm the status of *S. bertolonii,* together with the potential identification characteristics for differentiation from sage, both taxa were analyzed through the analysis of their essential oils, together with the micromorphological characteristics of the leaf surface and the anatomy and morphology of the leaves. The obtained results show that there are clear differences in the quantity of essential oil (0.073% for *S. pratensis* and 0.0016% for *S. berolonii*). The major compound in the essential oil of *S. pratensis* was *E*-caryophyllene (26.4%) while in *S. berolonii* essential oil caryophyllene oxide was the major component (35.1%). The micromorphological differences are also pronounced in the leaf indumentum (density and distribution of certain types of non-glandular and glandular trichomes). Clear distinction between the investigated *Salvia* species is also observed in the leaf anatomy (in *S. pratensis* leaves are thinner, palisade tissue is made of 1-2 layers of cells, and leaves of *S. bertolonii* are characterized by 2-3 layers of palisade tissue cells, and consequently thicker).

## Introduction

*Salvia*, the largest genus of Lamiaceae, includes about 900 species, widespread throughout the world. In Flora Europaea 36 taxa are described [1]. Some members of this genus such as sage (*Salvia officinalis* L.), balsamic sage (*S. tomentosa* Miller) and greek sage (*S. triloba* L.) are of economic importance, since they are used as flavoring agents in perfumery and cosmetics, but all of these species have been credited with a long list of medicinal uses: e.g. spasmolytic, antiseptic, astringent [2]. Some of the essential oils and phenolic compounds of plants belonging to this genus have also shown excellent antimicrobial activity as well as antioxidant capacity [3, 4] and consequently, the corresponding extracts have been widely used to stabilize fat and fat-containing foods [5].

Many of wild growing *Salvia* species are sometimes used in traditional medicine of different nations instead of sage or as an adulteration, because of very similar surface and shape of leaves [6]. Furthermore, the differences in biological activities of the drug could be observed, related to the different compounds present in plant material used [7]. Two of these plants are *Salvia pratensis* L.and *S. bertolonii* Vis. [8]. However, these taxa are described under one synonym as *S. pratensis* L in Flora Europaea [1].

With respect to this, in the present paper the essential oils of *Salvia pratensis* L. and *S. bertolonii* Vis. were chemicaly caracterized, together with the micromorphological studies of indumentum at leaves (types and density of glandular hairs) and leaf anatomy and morphology, with the purpose of clarfying the possible relationship between these spp.

## Results and Discussion

### Chemical composition of the essential oils

The mean content of the essential oil in the leaves expressed in percentages for *S. pratensis* was 0.073% and for *S. bertolonii* 0.0016% v/w dry matter. According to the earlier published data [9, 10], among 50 *Salvia* species only a few (*S. officinalis*, *S. grandiflora*, *S. triloba* and *S. sclarea*) have a notable quantity of essential oil, and the majority of species contain only traces of the oil. Although the yield of the essential oil in both of the examined species permits the assignment to the oil-poor group of the *Salvia* genus, the quantity of the essential oil was notably higher in *S. pratensis*. The percentage composition of the essential oils of *S.pratensis* and *S.berolonii* is presented in Table 1.

**Table 1:** Chemical composition (%) of essential oils of *S. pratensis* and *S. bertolonii*.

Peak No.	Components	R.I.^a^	*S. pratensis*	*S. bertolonii*	Identification method^ b^
	**Monoterpene hydrocarbons**		**0.3**	**0.2**	
1	*α-*Pinene	935	traces	0.1	GC-MS
2	Camphene	956	0.1	-	MS
3	Sabinene	972	0.1	-	GC-MS
5	Limonene	1032	0.1	-	GC-MS
8	*γ-*Terpinene	1060	-	0.1	GC-MS
	**Oxygenated Monoterpenes**		**1.1**	**7.2**	
6	1,8-Cineole	1034	0.4	-	GC-MS
7	Benzene acetaldechyde	1041	0.1	-	MS
9	Phenol, 2-(1*Z*)-propenyl	1150	0.2	-	MS
10	Borneol	1169	-	4.0	GC-MS
11	1-*α-*Terpineol	1188	0.2	1.2	GC-MS
12	Methyl chavicol	1196	-	2.0	GC-MS
14	Phenol, 2-(1*E*)-propenyl	1266	0.2	-	MS
	**Sesquiterpene hydrocarbons**		**53.7**	**21.9**	
16	*α-*Cubebene	1348	0.2	-	MS
17	Cyclosativene	1372	0.1	-	MS
18	*α-*Copaene	1378	0.2	-	MS
19	*β-*Cubebene	1390	5.6	0.4	MS
20	*β-*Elemene	1392	1.4	-	MS
21	*Z*-Caryophyllene	1405	0.2	11.4	MS
22	*E*-Caryophyllene	1419	26.4	2.9	GC-MS
23	Aromadendrene	1441	0.8	-	MS
25	*α-*Humulene	1452	2.9	3.3	GC-MS
26	*γ-*Gurjunene	1476	0.5	-	MS
27	*epi*-Bicyclo sesquiphellandrene	1490	5.6	-	MS
29	*γ-*Cadinene	1514	-	1.2	MS
30	*δ-*Cadinene	1524	0.4	-	MS
31	*Z*-*β-*Farnesene	1526	6.0	2.7	MS
32	Germacrene B	1562	3.4	-	MS
	**Oxygenated Sesquiterpenes**		**1.4**	**35.1**	
33	Spathulenol	1578	0.8	-	GC-MS
34	Caryophyllene-oxide	1582	-	35.1	MS
37	Vulgarone B	1658	0.6	-	MS
	**Aliphatic Components**		**15.7**	**10.8**	
4	Nonanal	1101	1.3	3.0	MS
13	Dodecane	1197	-	1.6	MS
15	Cyclodecane		-	2.1	MS
24	2-Pentadecanone		4.1	3.7	MS
28	Pentadecane		0.5	-	MS
35	Hexadecane		0.5	-	MS
36	Tetradecanal		0.8	-	MS
38	Tetradecanoic acid		0.6	0.1	MS
39	Heptadecane		0.9	-	MS
40	1,3,6-Heptatriene, 2,5,5-trimethyl		1.0	-	MS
41	Octadecane		0.3	-	MS
42	Hexadecanol		0.8	-	MS
43	Nonadecane		0.3	0.1	MS
44	Hexadecanoic (palmitic) acid		0.3	-	MS
45	Hexadecanoic acid, 1-methyl ethyl ester		1.9	-	MS
46	Heneicosane		0.4	-	MS
47	Heptacosane		0.7	-	MS
48	Octacosane		0.7	0.2	MS
49	Tetratriacontane		0.6	-	MS
	**Amount of identified compounds**		**72.2**	**74.3**	

^a^ Retention indices relative to C_9_-C_24_ n-alkanes on the HP 5MS column; ^b^ GC-identification based on retention times of authentic compounds on HP 5MS column; MS-tentatively identified on the basis of computer matching of the mass spectra of peaks with the NIST/NBS and Wiley libraries and those reported by Adams [12]

For *S.pratensis* a total of 42 chemical constituents, representing 72.2% of the total content, were identified. On the other hand, in *S.bertolonii* 19 compounds were identified, representing 74.3% of the total essential oil content. The major class of substances in the essential oil of *S. pratensis* was the sesquiterpene hydrocarbons group (53.7%), followed by aliphatic compounds (15.7%). On contrary, oxygenated monoterpenes were found to be the major class of substances (35.1%) in the essential oil of *S. bertolonii*, followed by sesquiterpene hydrocarbons (21.9%) and aliphatic compounds (10.8%).

The main compound in *S. pratensis* essential oil was *E*-caryophyllene (26.4%), and this finding is in accordance to the earlier published data [10]. The following major compounds of the essential oil were epi-bicyclosesquiphellandrene (5.6%), *Z*-*β*-farnesene (6.0%) and *β*-cubebene (5.6%). Notable qualitative differences in the chemical composition of the essential oil of *S. pratensis* were observed compared to the essential oil of *S. bertolonii* (Table 1), in which caryophyllene oxide (35.1%) was the major component, followed by *Z*-caryophyllene (11.4%), *a*-humulene (3.3%) and two monoterpene alcohols, borneol (4.0%) and methyl chavicol (2.0%).

From the chemosystematic point of view, the main constituents of the investigated essential oils (*E*-caryophyllene in *S. pratensis* and caryophyllene oxide in *S. bertolonii*) can be used as the stable chemosystematic markers in the taxonomy of these two species. Also, the high content of one or both of these two compounds in the essential oil could be used as an identification character for adulteration of sage, in which camphor, *α*- and *β*-thujone are the main constituents [4].

### Leaf morphology and anatomy

Rosette and stem leaves of *S. pratensis* are pointed at the apex, with dentate leaf margins, rarely double dentate. On contrary, leaves of *S. bertolonii* are rounded at the apex, with double dentate leaf margin and shallowly lobed (Figure 1a and Figure 1b).

**Figure 1a:**
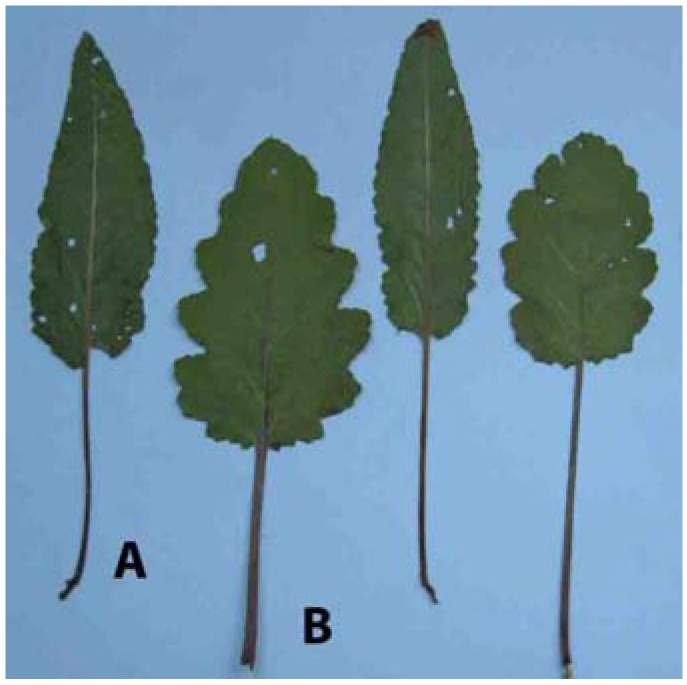
Shapes of the rosette leaves in *S. pratensis* (A) and *S. bertolonii* (B).

**Figure 1b:**
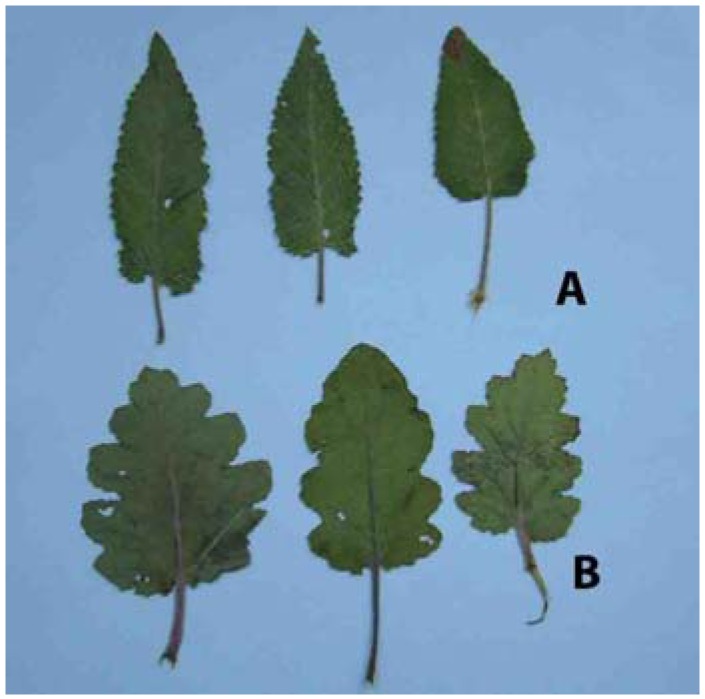
Shapes of the stem leaves in *S. pratensis* (A) and *S. bertolonii* (B).

**Figure 2:**
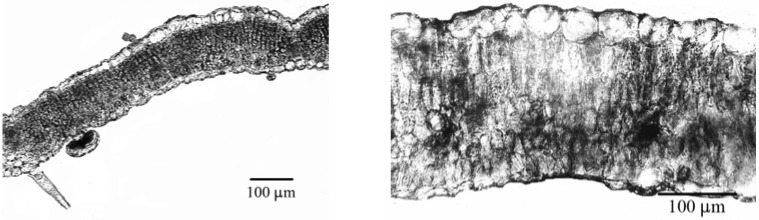
Leaf cross section of *S. pratensis* (left) and *S. bertolonii* (right).

Clear distinction between these two species is also observed in leaf anatomy (Figure 2). Compared to *S. bertolonii*, *S. pratensis* leaves are thinner and palisade tissue is made up of 1-2 layers of cells (Table 2a). Abaxial epidermal cells are smaller and with thinner cuticle. Stomata are more numerous, smaller and similar in size on both epidermises (Table 2b). Leaves of *S. bertolonii* are characterized by 2-3 layers of palisade tissue cells and consequently thicker. Epidermal cells are larger, with thicker cuticle (Table 2a). In this species, stomata are less numerous and larger, compared to *S. pratensis* (Table 2b).

**Table 2a:** Leaf anatomy characteristics^a^ of *S. pratensis* and *S. bertolonii.*

Characteristics			*S. pratensis*	*S. bertolonii*
Leaf thickness			146.5 ± 17.0	250.0 ± 23.0*
Mesophyll thickness			90.7 ± 8.3	217.1 ± 15.8*
Palisade tissue cells		Height	26.2 ± 5.8	48.0 ± 11.3*
		Width	13.7 ± 2.9	12.9 ± 2.7
Epidermal cells	Adaxial	Height	35.2 ± 8.7	39.9 ± 10.2*
		Width	40.8 ± 8.0	46.0 ± 9.4*
		Cuticle	3.8 ± 0.2	5.1 ± 0.3*
	Abaxial	Height	20.0 ± 3.2	24.5 ± 6.0*
		Width	25.8 ± 4.4	39.0 ± 7.9*
		Cuticle	2.7 ± 0.2	3.6 ± 0.3*

^a^all measurements are presented in µm *differences were statistically significant at p≤0.05

### Micromorphological features

The results of scanning electron microscopy (SEM) analysis of leaf indumentum indicate that both species are characterized by glandular and non-glandular trichomes. The differences in indumentum include only density and distribution of certain types of non-glandular and glandular trichomes (Figure 2). On both epidermal sides of *S. pratensis* leaves capitate trichomes (stalk composed of 1-2 and head of 1-2 cells) are present. The glandular trichomes of second type, peltate ones, appear only on the abaxial epidermis. They are bicyclic, with secretory head composed of 8-12 cells. On contrary, *S. bertolonii* leaves possess shorter capitate trichomes on both epidermal sides, built from one stalk cell and one (rarely two) cells of the head.

**Table 2b:** Number and size of leaf stomata in *S. pratensis* and *S. bertolonii.*

Characteristics			*S. pratensis*	*S. bertolonii*
Number of stomata (mm^2^)	Adaxial epidermis		148 ± 24.6	140 ± 20.2
	Abaxial epidermis		390 ± 93.6	270 ± 32.9*
Stomata size (mm)	Adaxial epidermis	Length	27.4 ± 4.7	31.1 ± 5.7*
		Width	17.2 ± 1.6	20.5 ± 2.2*
	Abaxial epidermis	Length	24.8 ± 4.0	29.9 ± 5.5*
		Width	17.4 ± 1.6	20.6 ± 1.9*

Considering glandular trichomes, the main difference between these two species is that *S. pratensis* has peltate trichomes on abaxial epidermis, which are not recorded on *S. bertolonii* leaves, and more types of capitate trichomes. Therefore, higher essential oil content in *S. pratensis* leaves, compared to *S. bertolonii*, could be explained, at the first place, by the presence of peltate trichomes, which are typical the most responsible for essential oil production.

**Figure 3:**
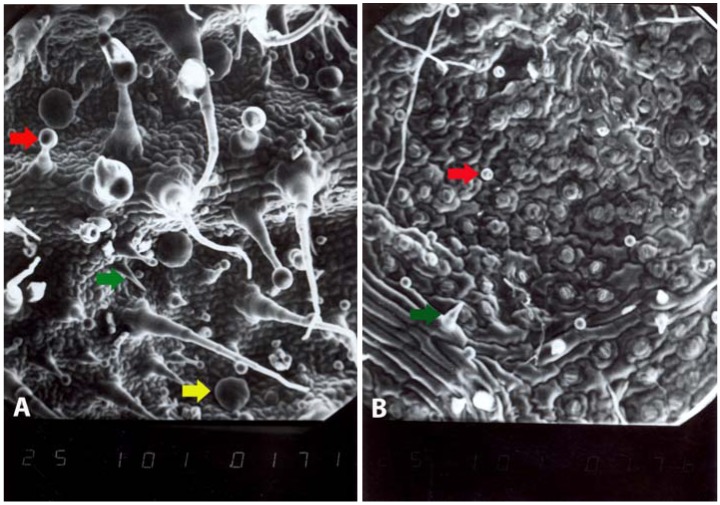
Glandular and non-glandular trichomes on abaxial epidermis of *S. pratensis* (A) and *S. bertolonii* (B) leaf. Non-glandular trichome (green), capitate glandular trichome with a short stalk (red), peltate glandular trichome (yellow).

In conclusion, the obtained results of both the chemical composition of the essential oils, and the micromorphological and anatomical features of the investigated *Salvia* species clearly distinguish these two taxa into two separate species (*S. pratensis* and *S. bertolonii*), confirming the earlier published data which clarify the uncertain taxonomic status of *S. bertolonii* [8].

## Experimental Section

### Plant material

*Salvia pratensis* L. was collected in full blossom at Fruška Gora mountain (Vrdnik, UTM: 34T DQ 1 09), Serbia, in May of 2007. *S. bertolonii* Vis. originated from Valdanos, near Ulcinj (Valdanos, UTM: 34T CM 4 54), Montenegro, collected in the same phenophasis, also in May of 2007. Voucher specimens of collected plants (*S. pratensis* no. 2-1898 and *S. bertolonii* no. 2-1899) were confirmed and deposited at the Herbarium of the Department of Biology and Ecology (BUNS), Faculty of Sciences, University of Novi Sad, Serbia.

### Isolation and analysis of the essential oil

Air-dried flowering tops in full blossom were submitted to hydrodistillation according to Eur. Pharm. 4 [11], using *n*-hexane as the collecting solvent. The solvent was removed under vacuum and the quantities of the essential oils were determined gravimetrically. Qualitative and quantitative analyses of the essential oils were carried out using a Hewlett-Packard 5973-6890 gas chromatography-mass spectrometry (GC-MS) system, operating in EI mode at 70 ev, equipped with a split-splitless injector (200^0^C) and a flame ionization detector (FID) (250^0^C). Helium was used as carrier gas (1 mL/min) and the capillary columns used were a HP 5MS (30 m x 0.25 mm; film thickness 0.25 򰂵m). The temperature programmes were 60°C to 280°C at a rate of 3°C/min and 60-260^°^C at a rate of 3°C/min, respectively; split ratio, 1:10. Coelution and MS analysis based the identification of individual compounds on comparison of their relative retention times with those of authentic samples (Carl Roth Gmbh; Karlsruhe, Germany). For the components, mostly sesquiterpenes and aliphatic compounds, for which reference substances were not available, the identification was performed by matching their retention indices and mass spectra with those obtained from authentic samples and/or the NIST/NBS, Wiley libraries spectra as well as with literature data [12].

### Morphoanatomical measurements

For morphological analysis 50 plants were separated and leaf margin and leaf apex were analyzed. For anatomical analysis cross sections of middle parts of lamina were made. Measurements of tissues and cells were taken on 30 plant samples of both species, and mean values ± SD were calculated. Stomata size and number were analyzed on epidermal prints [13]. All anatomical analyses were performed on a MOTIC 2000 Image Analysis system.

### Micromorphological studies

Morphology of trichomes was analyzed using SEM JEOL JSM 6460 LV.
